# Evidence for the Continued Occurrence of Chorioretinopathy in Working Sheep Dogs in New Zealand in 2010

**DOI:** 10.3390/ani11082229

**Published:** 2021-07-29

**Authors:** Adam B. O’Connell, A. Craig Irving, Paul L. Hughes, Naomi Cogger, Boyd R. Jones, Kate E. Hill

**Affiliations:** 1 Roseworthy Campus, The School of Animal and Veterinary Science, University of Adelaide, Adelaide, SA 5371, Australia; adam.oconnell@adelaide.edu.au; 2 EyeVet Services, 25 Manchester Street, Feilding 4472, New Zealand; craig@eyecarevet.com; 3 Taihape Veterinary Services, Kotare Street Taihape, Rangitikei 4720, New Zealand; prhughes@xtra.co.nz; 4 School of Veterinary Sciences, Massey University, Private Bag 11 222, Palmerston North 4442, New Zealand; N.Cogger@massey.ac.nz; 5 Working Dog Centre, Massey University, Private Bag 11 222, Palmerston North 4442, New Zealand; B.Jones@massey.ac.nz

**Keywords:** chorioretinopathy, chorioretinitis, working farm dogs

## Abstract

**Simple Summary:**

Previous research in New Zealand on working sheep dogs has found that 39% suffered from retinal eye disease. One of the causes of this eye disease is parasite migration to the eye. Since that research was published in 1987, farmers have been educated to regularly treat farm dogs with anti-parasitic tablets. However, there has been no follow-up studies to see if eye disease is still a problem in working farm dogs. Our study investigated whether eye disease is still present in working sheep dogs in South-West, Waikato, New Zealand. Our study of 184 working sheep dogs and 51 owners was undertaken in 2010, with owners sampled from New Zealand’s South-West Waikato and Tux North Island Dog Trial Championship. Eye examinations were performed on all of the dogs that participated. This study identified that 24% (44/184) of the dogs in the study showed evidence of retinal eye disease. Male working sheep dogs are more susceptible to develop retinal eye disease than females for unidentified reasons. This study concluded that retinal eye disease is still present in working sheep dogs in New Zealand.

**Abstract:**

A study in conducted 1987 by Hughes et al., found that 39% of working sheep dogs had multifocal retinitis. One of the identified causes was ocular larval migrans, which were a result of migrating ascarid larvae. Since that paper was published, anthelmintic use in farm dogs has been highly recommended. There has been no follow-up study to determine if fundic lesions are still present. The current study aimed to investigate the prevalence of chorioretinopathy in working sheep dogs in the South-West, Waikato, New Zealand. This was a cross-sectional study of 184 working sheep dogs and 51 owners, undertaken in 2010 with owners sampled from New Zealand’s South-West Waikato and Tux North Island Dog Trial Championship. Two-way tables were used to explore the relationship between variables. Significance of association was assessed using a Chi-squared or Fisher exact test as appropriate, with a *p*-value of <0.05 considered significant. Overall prevalence of chorioretinopathy in the working sheep dogs was 44/184 (24%). A significantly higher prevalence of chorioretinopathy was shown in dogs with increasing age, from 2 years to >8 years (*p* = 0.0007) and in males (*p* < 0.0001). This study concluded that lesions of chorioretinopathy are still present in working sheep dogs in New Zealand.

## 1. Introduction

There are a range of recognised pathological conditions that affect the canine fundus, including diseases that have been described in specific breeds [1,2,3]. A previous study by Hughes et al. (1987) [4] identified that multifocal retinitis was present in working sheep dogs; the authors observed that retinitis was more prevalent in the study population of working sheep dogs (39% of 1448 dogs) than in the population of urban dogs examined by the authors for comparative measures (6% of 125 dogs). However, pet dogs in a rural environment had a retinitis prevalence of 43% (30/70 dogs), thus portraying a similar risk as their working counterparts. Furthermore, these authors identified that ocular larval migrans, due to migrating ascarid larvae, was one cause of the retinitis and that *Toxocara canis* was the likely species involved. Since that paper was published, there have been recommended changes in husbandry (e.g., elevated motel style dog kennels) and anthelmintic use in farm dogs has been highly recommended; most rural practices ensure that their farmer clients undertake anthelmintic treatment of their dogs. There has been no subsequent examination of any population of working dogs to determine if fundic lesions are still present. Thus, the aims of this study were to investigate whether evidence for chorioretinopathy was still present in working sheep dogs, to determine its prevalence, and to associate the prevalence of chorioretinopathy with age, gender, breed, and *Toxocara canis* infection status. In this paper, the terms chorioretinitis (inflammation affecting the choroid and adjacent retina) and chorioretinopathy (any lesion of the choroid and retina including non-inflammatory causes) are used in preference to retinitis and retinopathy as the close proximity and functional intimacy of the retina and choroid mean that inflammation of one of these tissues will normally lead to inflammation of the other, resulting in chorioretinitis or retinochoroiditis [5].

## 2. Materials and Methods

This was a prospective study that was approved by the Massey University Animal Ethics Committee MUAEC 10/10.

Fifty-one owners of working dogs in the Waikato region and competitors in the Tux 2010 North Island Dog Trial Championship in Gisborne were convenience sampled and 184 dogs enrolled in the study. Data was collected between the 11 April 2010 and 7 July 2010.

The owners and their dogs were convenience sampled based on their proximity to the veterinary clinic and other farms being sampled, the number of dogs available at the farm location, the ease of establishing contact with the working dog owner, willingness of the owner to participate and the ability to arrange a time for sampling that suited both the owner and the investigator. The criterion for initial inclusion into the study was that the owner and their dog(s) worked in a sheep farming system and that the dog(s) were greater than six months of age. For the purpose of this study, the definition of a sheep farming system was a commercial sheep-rearing facility located on an extensive area of land. The 15 dogs examined at the dog trial were volunteered by their owners. None of the dogs had known problems with their vision.

All of the dogs were six months of age or older and both the owner and the dog worked on a commercial sheep farm at the time of the study. Details of the dogs’ ownership and information about the husbandry of the dogs was collected by a questionnaire. Faecal samples were collected from 170 dogs. The samples were stored at 4 °C from 1 to 30 days prior to the parasitological examination. A zinc-sulphate centrifugation faecal- float technique was completed for all dogs in which faecal samples were obtained and the ova of *Toxocara canis* was identified when present. The procedure used for the faecal examination was based on the flotation method described by Zajac, Johnson and King (2002) [6]. For the extended methodology and results of the fecal analysis, please refer to O’Connel et al. 2019 [7].

All fundic examinations were performed by the same veterinary field investigator (AO). The field investigator was trained by an Ophthalmology specialist (ACI) to use and be skilled in the use of indirect and direct ophthalmoscopes, data recording with the Optibrand fundic camera and to detect retinal lesions. A mydriatic (one to two drops), containing the active ingredient tropicamide (Mydriacyl^®^ 1%, Alcon Laboratories, Auckland, New Zealand), was applied to the conjunctivae of each eye of all the working sheep dogs before any other sampling activity was undertaken. Menace, dazzle and palpebral eye reflexes were observed after the mydriatic was applied. The prior administration of the mydriatic meant that pupillary light reflexes (PLR) were not tested. The orbit, adnexa, cornea, iris, and lens were closely examined using natural and artificial light (pen torch). An indirect ophthalmoscope with a 20-dioptre lens was used to examine the lens, vitreous and fundus in a convenient shaded or dark area. Abnormal or atypical fundi were photographed using the Clearview Optibrand Fundic Camera. The fundic images were downloaded to a laptop computer and stored using proprietary software for Clearview Optibrand fundic images.

Once all the dogs had been examined, the veterinary field investigator and two co-authors examined the fundic images. The final diagnosis was based on the images and the notes made at the time of the fundic examination. Fundi were identified as being either normal or diseased (chorioretinopathy). Normal fundi were not photographed.

The lesions of chorioretinopathy were described by the presence of six possible clinical signs, often with two or more signs occurring simultaneously:

1. Multifocal reflective changes
2. Focal reflective areas
3. Multifocal pigmentary changes
4. Focal pigment deposition
5. Blood vessel attenuation
6. Optic nerve atrophy

A particular chorioretinopathy might be described as having ‘focal-reflective changes, focal pigment deposition, blood vessel attenuation’.

Data analyses were performed in Microsoft Excel 2010 and the statistical software package R Version 2.13. Initially, datasets were examined for completeness and validity.

The prevalence of chorioretinal disease was described using both a count of the number of normal and of diseased dogs and calculated as a proportion of total number of dogs who had their fundi examined. The distribution of chorioretinopathy between the left eye and the right eye was described using a count of the number of eyes affected. The six clinical signs supportive of chorioretinal disease used in this study were described using a count of the number of dogs with clinical signs of interest and as a proportion of the total number of dogs who had either a normal fundus or a diseased fundus, which was imaged and reviewed. Prevalence of *Toxocara canis* was described using both a count of the number of normal and the number of diseased dogs and as a proportion of all the dogs who underwent a faecal examination. Two-way tables were constructed to explore the relationship between chorioretinal disease (lesion or no lesion) and age, gender, breed, and *Toxocara canis* infection status, as well as by *Toxocara canis* infection status and age, and the relative risk of chorioretinal disease with 95% confidence intervals. The significance of the associations were assessed using a Chi-squared or Fisher-exact test, as appropriate. A *p*-value of <0.05 was considered statistically significant.

## 3. Results

All the dogs with retinal lesions detected by the primary investigator (AO) were confirmed to have lesions on specialist review. Table 1 shows the number and relative frequency of the six signs of chorioretinal disease, classified in this study by each dog that was imaged for further assessment. One hundred and eighty-four dogs had both eyes examined. The prevalence of chorioretinal disease in the working sheep dogs was 44/184 dogs (24%). A total of 31 left eyes had chorioretinopathy, while the number of right eyes with chorioretinopathy was 36. Twenty-three dogs had bilateral fundic lesions. There was no statistically significant difference in the frequency of clinical signs of chorioretinal disease between the right eye and left eye (*p* = 0.79).

**Figure 1:**
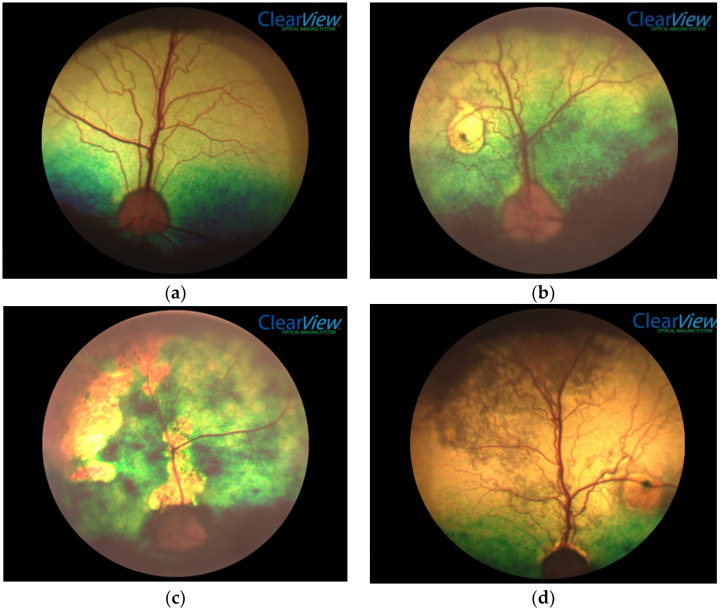

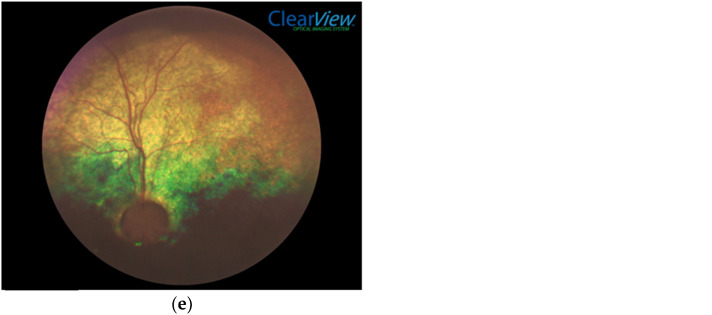
Examples of a normal fundus and six clinical signs of chorioretinal disease observed in a study of chorioretinopathy in working sheep dogs in New Zealand. (**a**) Example of a typical normal fundus for a working sheep dog. (**b**) Example of a focal fundic lesion showing focal hyper-reflectivity and focal pigment deposition in a working sheep dog. (**c**) Example of diffuse fundic lesions showing diffuse hyper-reflectivity and diffuse pigment deposition, blood vessel attenuation and optic nerve atrophy in a working sheep dog. (**d**) Example of focal pigment deposition, focal reflective change, diffuse hyper-reflectivity and diffuse pigment deposition, blood vessel attenuation and optic nerve atrophy in a working sheep dog. (**e**) Example of optic nerve atrophy, blood vessel attenuation, diffuse hyper-reflectivity, and diffuse pigment deposition in a working sheep dog.

The lesions of chorioretinopathy ranged from small focal and discrete lesions to diffuse or multifocal and poorly delineated lesions involving the whole fundus (see Figure 1a–e). The tapetal fundic lesions were typically hyper-reflective with or without pigment deposition, while non-tapetal fundic lesions typically had a patchy loss of pigment deposition and were paler in colour. Retinal blood vessels were often attenuated and optic nerve atrophy, the least common finding, was only found in association with other evidence of chorioretinopathy.

Loss of vision, assessed by menace and dazzle reflex, was absent in all dogs with chorioretinopathy and none of the owners identified problems with their dog’s vision. A small number of palpebral, orbital, adnexal, corneal, anterior chamber, iris and lens abnormalities were found but none appeared to have any relationship with the chorioretinal disease if they occurred with the fundic lesions. No vitreal lesions were found.

One hundred and fifty-three working sheep dogs underwent faecal examinations and fundic examinations. A totoal number of 9 dogs out of the 153 (6%) examined had *Toxocara canis* ova in their faecal samples.

Complete results of the faecal examinations and anthelmintic therapy are found in O’Connell 2019 [7].

Table 2 shows the relationship between chorioretinal disease and age, breed, gender, and *Toxocara canis* infection status. There was a statistically significant relationship between the presence of chorioretinal disease and age (*p* = 0.0007). While chorioretinal disease occurred in all age groups, the prevalence did increase with increasing age. Dogs aged eight years old or older were 6.3 times more likely to have chorioretinopathy than dogs under two years of age. There was also a statistically significant relationship between chorioretinal disease and gender, with males having a higher prevalence of chorioretinal disease than females (*p* < 0.0001).

There was no statistically significant relationship found between chorioretinal disease breed, and *Toxocara canis* status in dogs that had positive egg counts.

## 4. Discussion

The current study confirmed that chorioretinal disease is still present in New Zealand’s sheep dogs and that the prevalence in the Waikato district was 24%. None of the dogs examined at the National trial had ocular lesions. The prevalence in the current study is lower than that identified by Hughes et al. (1987), who found 39% of dogs in the Taihape region and at national dog trials had multifocal retinal disease on direct ophthalmoscopic examination. No current data on the prevalence of chorioretinal disease in urban dogs is available for comparison, but the prevalence found in this current study is considerably higher than the prevalence of 6% of urban dogs with retinitis identified by Hughes et al. (1987). The lower prevalence in working sheep dogs in this current study could be due to chance or could reflect a reduction in the prevalence of chorioretinal disease due to improvements in the ‘on farm’ anthelmintic programmes and the efficacy of these anthelmintics. However, it could reflect other unidentified causes. Alternatively, the geographical, husbandry and climatic differences in the location of the working sheep dog population in this study compared to the study of Hughes et al. (1987) may have resulted in a lower prevalence of chorioretinal disease in the current study. Both studies may be under-reporting the prevalence of the disease due to peculiar environmental conditions associated with the on-site research, in particular sub-optimal lighting conditions for thorough eye examinations and inadequate restraint of the dogs by owners inexperienced in restraining animals for eye examinations.

Chorioretinal lesion(s) are not typically pathognomonic for any particular disease and other tests are required to identify a cause. However, lesions consistent with chorioretinitis (see Figure 1b–e) can be confused with other ocular diseases in dogs. Previous studies in pet dogs as well as the one study in working dogs in NZ have documented that *Toxocara canis* was a cause of chorioretinitis [4,8,9]. The lesions seen in this study could be a consequence of *Toxocara canis* or of other diseases; the latter causes were not investigated. A small number of dogs in the current study (9/153, 6%) were infected with *Toxocara canis*. A previous study by the same authors has shown that more than 80% of the dogs were reportedly given anthelmintic drugs at least every three months; however, providing drugs at this frequency did not eliminate *Toxocara canis* infection. The results suggest that farmers should not rely on anthelmintic administration as the sole method of gastrointestinal parasite control in their working sheepdogs. Further research is needed to determine whether current treatment regimens are appropriate [7].

In the current study, no dog appeared to have loss of vision despite some having severe chorioretinal lesions. A recent study of the health of 641 working dogs in the South Island of NZ included 7 dogs with vision loss or deficits. A fundic examination was not part of the physical examination protocol in that study, so no potential cause was found [10]. In the previous study by Hughes, owners reported 4% (8/211) of dogs to be visually impaired [4]. The results from our study indicate that severe chorioretinal disease causing blindness has decreased since Hughes’ study in 1987.

Both the current study and that of Hughes et al. (1987) found a significantly higher prevalence of chorioretinal lesions in older working sheep dogs. The prevalence of diseases like chorioretinopathy, that do not result in death but from which they do not recover will increase with age. Therefore, it was expected that older animals in the current study would have a significantly higher prevalence of chorioretinopathy.

In the current study, males had significantly higher prevalence of chorioretinal disease than females. Hughes et al. (1987) reported a similar finding in both heading dogs and huntaways but did not identify a reason for the gender difference. In this current study, differences between male and female huntaways and male and female heading dogs were not determined. Future studies could expand on gender as a risk for chorioretinal disease in working dogs.

We do not know when the chorioretinopathy occurred or if the animal was parasitised with *Toxocara canis* at the time the lesions of chorioretinopathy developed. The association of ocular lesions and *Toxocara canis* infection is accepted from Hughes et al’s study and our investigation confirms similar retinal lesions being present but no direct association shown.

None of the other variables investigated (see Table 2) were significantly associated with the prevalence of chorioretinal disease. The very small number of dogs found with other ocular diseases meant that no association between non-fundic ocular disease and chorioretinal disease was possible.

Applying a mydriatic prior to assessing ocular responses diminished the ability to assess one component of retinal function as observation of the non-medicated eye and of the pupillary light reflexes were not available. Application of mydriatic at the beginning of the eye examination was a necessary compromise due to the limited time available for farmer assistance when the on-farm survey and examination were being performed. The menace response, dazzle and palpebral reflexes were still tested in all the dogs.

The inclusion criteria for this study were very broad, with no attempt to define a commercial farm. Therefore, it is possible that some owners and their dogs were not bona-fide farmers, and their dogs may not have been working sheep dogs. The study was convenience sampled, which could cause non-observational bias, with owners able to exclude their dogs if they elected to. The majority of owners were from the same geographical area, with a sub-set of sheep dog triallists; furthermore, owners may treat their working sheep dogs differently from commercial farmers. Therefore, our results may not be representative of the general working sheep dog population. Observational bias may have arisen due to false answers when the questionnaire was being conducted as it was not procured as an anonymous questionnaire. Substantial random error potentially occurred due to the small sample size. A single investigator, who was a veterinarian, performed all examinations. The investigator was well trained and able to determine affected and healthy dog retinas. The authors are confident all affected dogs were identified.

In contrast to the study of Hughes et al. (1987) study, no histopathology of any ocular lesions was undertaken. Further pathological studies of the eyes of working sheep dogs, particularly those with active eye disease, might provide greater knowledge surrounding the additional causes of ocular lesions, including choirioretinitis in working dogs.

## 5. Conclusions

This study identified that 44/184 dogs had evidence of chorioretinal disease. The limited information provided in this study indicates the prevalence of chorioretinal disease may have decreased in working sheep dogs since 1987 (Hughes et al. 1987). For unidentified reasons, ale working sheep dogs are significantly more susceptible to develop chorioretinal disease than females.

## Figures and Tables

**Table 1:** Frequency of six clinical signs of chorioretinal disease in 184 working sheep dogs in a study of chorioretinopathy in a population of working sheep dogs in New Zealand.

Clinical Sign	Dogs Affected	Proportion of Total (*n* = 184)
Diffuse hyper-reflectivity	23	0.13
Focal hyper-reflectivity	20	0.11
Diffuse pigment deposition	25	0.14
Diffuse pigment deposition	18	0.10
Blood vessel attenuation	20	0.11
Optic nerve atrophy	13	0.07

**Table 2:** Association of age, breed, gender, and *Toxocara canis* infection on the prevalence of chorioretinal lesions in a study of chorioretinal disease in a population of working sheep dogs in New Zealand.

Variable	Status	Chorioretinopathy Positive Dogs	Chorioretinopathy Negative Dogs	Relative Risk of Chorioretinal Disease (95% CI)	*p*-Value
Age ^a^	<2 years old	2	32	REF	0.0007
	2–3 years old	6	42	2.13 ^e^ (0.46–9.90)	
	4–7 years old	22	42	5.84 (1.46–23.38)	
	≥8 years old	13	22	6.31 (1.54–25.9)	
Gender ^b^	Male	38	70	REF	<0.0001
	Female	6	69	0.23 (0.10–0.51)	
Breed ^c^	Heading	18	56	REF	0.96
	Huntaway	20	59	1.04 (0.60–1.81)	
	Crossbreed	5	23	0.73 (0.3–1.79)	
Toxocara canis ^d^ status	Infected Uninfected	2 30	7 114	REF 0.94 (0.27–3.2)	0.74

Data missing for ^a^ 3 cases, ^b^ 1 case, ^c^ 3 cases, and ^d^ 31 cases. Dogs were excluded if they did not undergo a faecal examination or the owner of the dog did not complete the relevant section of the questionnaire. ^e^ The relative risk of chorioretinal disease in working sheep dogs aged 2–3 years was 2.13 (95% CI 0.46–9.90) times more than those working sheep dogs with an age of less than two years. REF—When calculating relative risk, REF (reference) is the value that was used for comparison with the other relevant values.

## Data Availability

The data is available from the corresponding author upon request.
